# Intraoperative Diagnosis of Sodium-Glucose Transporter-2 Inhibitor-Associated Euglycemic Diabetic Ketoacidosis

**DOI:** 10.7759/cureus.71931

**Published:** 2024-10-20

**Authors:** Bibek Devkota, Timothy Maxwell, Jessica Schaedel, Brant M Wagener, Weifeng Song, Nishank Patel Nooli

**Affiliations:** 1 Anesthesiology and Perioperative Medicine, University of Alabama at Birmingham Heersink School of Medicine, Birmingham, USA; 2 Anesthesiology and Critical Care, University of Alabama at Birmingham Heersink School of Medicine, Birmingham, USA; 3 Cardiac Anesthesiology, University of Alabama at Birmingham Heersink School of Medicine, Birmingham, USA

**Keywords:** euglycaemic diabetic ketoacidosis, high anion gap metabolic acidosis, perioperative diabetic ketoacidosis, pre-operative evaluation, sodium-glucose transporter-2 inhibitors

## Abstract

Sodium-glucose transporter 2 inhibitors (SGLT2i) are increasingly used in diabetic patients having cardiovascular and renal comorbidities. Despite their benefits for glucose control and reducing cardiovascular complications, they are not without risks. We present a case of euglycemic diabetic ketoacidosis (DKA) in a 60-year-old male with metastatic melanoma and type 2 diabetes mellitus (DM) on empagliflozin, undergoing craniotomy for brain tumor resection. Intraoperatively, high anion gap metabolic acidosis with normal blood sugar levels was observed, leading to the diagnosis of euglycemic DKA. Management included immediate initiation of intravenous insulin with dextrose, which was continued in the neuro-intensive care unit (NICU) postoperatively for three days. Euglycemic DKA is sometimes tricky to diagnose due to the absence of significant hyperglycemia as the name suggests, potentially delaying recognition by clinicians. Early detection, intravenous insulin with dextrose, correction of metabolic derangements, and discontinuation of SGLT2i are essential components of management. This case underscores the necessity of considering euglycemic DKA in SGLT2i-treated patients undergoing surgery, particularly when metabolic acidosis with a high anion gap is present despite normal blood glucose levels.

## Introduction

Sodium-glucose transporter 2 inhibitors (SGLT2i) represent a novel class of medications approved for managing type 2 diabetes mellitus (DM). These drugs enhance urinary glucose excretion alongside sodium, yielding promising outcomes in diabetic patients, particularly those burdened with cardiovascular issues. Recent trials have underscored their notable mortality benefit, reduction in HbA1c levels, blood pressure, weight, and heart failure-related readmissions [1]. Consequently, more surgical patients are now on SGLT2i therapy. Despite their cardiorenal benefits, SGLT2i have been linked to adverse events, including euglycemic DKA, urinary tract infections, acute kidney injury, and limb amputations [1-3]. The emergence of euglycemic diabetic ketoacidosis (DKA) post-SGLT2i introduction has been documented in various case reports and series, presenting a life-threatening emergency [4,5]. This condition, characterized by ketoacidosis and mild hyperglycemia with blood glucose levels <250 mg/dL, poses diagnostic challenges and mandates timely intervention to mitigate metabolic derangements [6]. Euglycemic DKA can arise in patients with both type 1 and type 2 DM, as well as due to various precipitating factors, including pregnancy, decreased caloric intake, alcohol abuse, insulin administration pre-hospitalization, and comorbidities such as pancreatitis, sepsis, and liver disease [7]. Treatment entails swift correction of dehydration, electrolyte imbalances, and insulin infusion until normalization of anion gap and bicarbonate levels. Moreover, higher dextrose concentrations (10 or 20%) may be necessary to facilitate concomitant insulin administration for severe acidosis resolution [6,7].

## Case presentation

A 60-year-old male, with malignant melanoma and metastatic brain lesions with a new frontal lobe lesion and type 2 DM on empagliflozin, was hospitalized after presenting the night before the scheduled craniotomy, with altered mental status (AMS) and expressive aphasia. Laboratory parameters done a week before during the pre-anesthetic visit showed normal glucose, bicarbonate, and anion gap with HbA1c of 6.7 and stable intracranial imaging. The patient was admitted to the neuro-intensive care unit (NICU) for preoperative assessment and optimization. Preoperative labs showed low bicarbonate levels (11), high anion gap (29), and normal blood glucose, which was overlooked. The case proceeded the next morning under general anesthesia, and the baseline arterial blood gas (ABG) showed metabolic acidosis. Initially, it was thought to be due to hypovolemia, with no response to supplemental fluid. After conducting a thorough differential diagnosis, we arrived at the potential diagnosis of euglycemic DKA, prompted by the patient's use of empagliflozin. A blood sample was immediately sent to check for basic metabolic panel and serum ketones. Subsequent intraoperative ABGs were done to monitor the electrolytes and metabolic acidosis (Table 1).

**Table 1:** Important laboratory values in the perioperative period NICU - Neuro-Intensive Care Unit

Hours from Induction of Anesthesia	pH	Glucose	Bicarbonate	Anion Gap	Beta-hydroxybutyrate	Base Excess
-16	-	110	20	20	-	-
-7	-	136	11	29	-	-
0	7.36		12	-		-11
1	7.20	126	13	-		-14
3	7.23	147	13	-		-13
4	7.25	115	12	20		-14
5	7.25	155	16	-		-10
6	7.27	151	17	-		-9
7	7.30	147	18	-	3.1	-8
Postoperative Day in NICU						
0	7.50	147	22	14	-	-2.2
1	7.44	109	25	11	3.2	-1.0
2	7.45	131	24	12	2.4	2.6
3	7.43	99	25	10	0.8	3.1

This conclusion was supported by the elevated beta-hydroxybutyrate levels, measured at 3.1 mmol/L. Treatment was empirically started after sending out a blood sample for measurement of ketones, with intraoperative insulin infusion and 5% dextrose with normal saline. The patient was transferred to NICU postoperatively, and DKA management was continued for three days till blood gases became normal and the anion gap closed.

## Discussion

**Figure 1:**
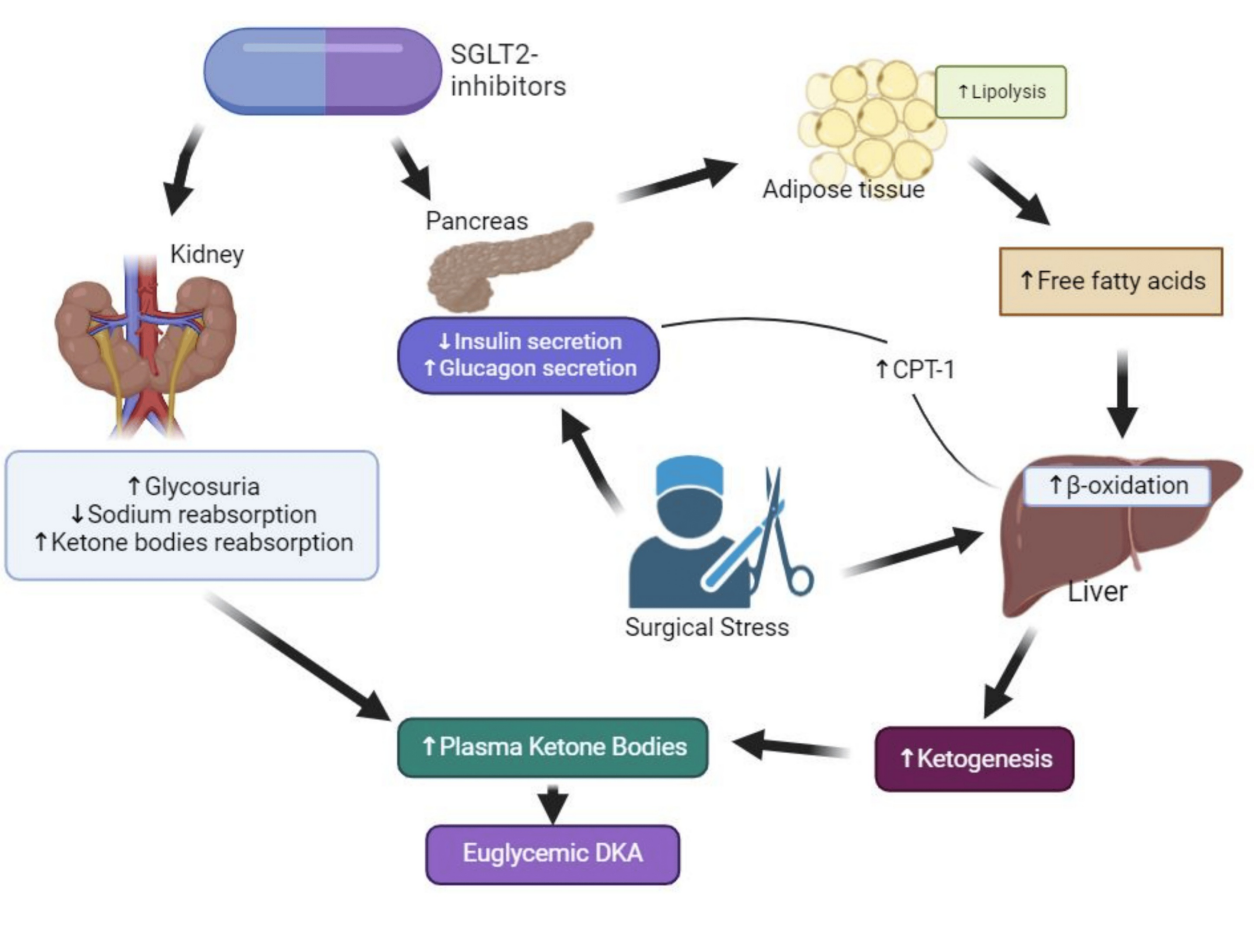
Pathophysiology of euglycemic DKA in patients taking SGLT2 inhibitors SGLT2 - Sodium Glucose Transporter-2; CPT I - Carnitine palmitoyl transferase I Sources: Refs. [17,18]

SGLT2i represent a recently approved class of antihyperglycemic medications by the Food and Drug Administration (FDA). They operate via a unique mechanism, reducing the reabsorption of glucose in the renal tubules, thereby lowering blood glucose levels without triggering insulin secretion. Additionally, they may offer advantages such as improving blood pressure and aiding in weight management. Their prescription is increasing significantly due to the improved outcomes observed in heart failure and chronic kidney disease linked with type 2 DM [8]. Reports of euglycemic DKA have increased with widespread use of SGLT2i, especially during the perioperative period [9-13]. The incidence of EDKA is about 0.17 % in non-emergent and 1.1% in emergent procedures. SGLT2i may cause euglycemic DKA through a variety of mechanisms. SGLT2i enhance glucose excretion in the kidneys and increase the reabsorption of ketone bodies, leading to relatively low insulin levels. In the pancreas, SGLT2i suppress beta cell activity, which stimulates lipolysis and further contributes to the pool of ketone bodies [14]. Direct effect on pancreatic α-cells results in increased glucagon secretion, leading to an imbalance of insulin/glucagon ratio. Increased lipolysis leads to heightened activity of carnitine palmitoyl transferase I (CPT I), facilitating beta-oxidation of fatty acids within mitochondria, and thereby inducing ketogenesis (Figure 1) [15,16].

Physicians must remain vigilant about diagnosing euglycemic DKA, as normoglycemia can mask and deter us from thinking of ketoacidosis, necessitating prompt initiation of treatment [16]. Reducing the incidence of SGLT2i-associated euglycemic DKA can be achieved through appropriate perioperative cessation of the medication, heightened clinical vigilance, and early detection through emphasis on serum beta-hydroxybutyrate levels [2]. Treatment involves swiftly addressing dehydration, correcting electrolyte imbalances, and administering insulin via drip until normalization of anion gap and bicarbonate levels is achieved [7,19,20].

During the perioperative phase, various physiological disruptions occur, such as dehydration due to fasting, restricted oral intake postsurgery, and heightened metabolic requirements, leading to a tendency toward ketosis. Additionally, surgical stress triggers the release of catecholamines, which in turn stimulate gluconeogenesis, lipolysis, and ketone production [1]. Before the surgery, our patient fasted for eight hours, consuming only a sip of water with their morning medications. The extensive surgery imposed considerable physiological strain on the patient, spanning over nine hours. The patient had taken their SGLT2i one day prior to the surgery. This patient presented a day before surgery with altered mental status, so we were unable to hold the medication for more than 24 hours. The FDA has recommended discontinuing canagliflozin, dapagliflozin, and empagliflozin three days before elective surgery, and ertugliflozin four days before elective surgery, owing to the potential risk of perioperative euglycemic diabetic ketoacidosis [10]. The manifestation of euglycemic diabetic ketoacidosis during intraoperative periods varies notably from its presentation in clinical settings. General anesthesia masks numerous signs and symptoms typically associated with ketoacidosis [13]. The patient's inability to express symptoms such as thirst, nausea, or lethargy during the intraoperative period renders the detection of euglycemic diabetic ketoacidosis challenging. In this instance, the sole indication of intraoperative euglycemic DKA was the development of an anion gap metabolic acidosis, which worsened despite appropriate crystalloid and electrolyte replacement, stable hemodynamics, and the absence of substantial blood loss. We considered a myriad of possibilities when evaluating the high anion gap metabolic acidosis. However, our suspicion leaned towards ketoacidosis because of SGLT2i therapy. The patient's serum beta-hydroxybutyrate concentration exceeded the normal limit by nearly two standard deviations, affirming the presence of ketoacidosis despite the serum glucose level being within the normal range. The altered mental status with high AGAP (20) and moderately decreased bicarbonate level (20) in the preoperative period with stable neuroimaging might be the first clinical sign of ketoacidosis, which probably was overlooked because of normal blood glucose and HbA1C and less awareness of this rare side effect of SGLT2i among the clinicians.

Due to the awareness of this rare but potentially lethal adverse effect of this drug, the NICU team at the author's institute was able to diagnose and manage another case of perioperative euglycemic DKA on time. A 65-year-old man with hypertension, type 2 DM on dapagliflozin, and a parafalcine meningioma presented with worsening left-sided weakness one day prior to his scheduled surgery. Due to his deteriorating neurological status despite optimal medical management, the surgery was rescheduled to an earlier date. His SGLT2i was discontinued just one day before surgery. Given previous experiences with intraoperative euglycemic DKA, we proactively addressed the potential risk of euglycemic DKA and metabolic abnormalities. Preoperative investigations were normal. During the 11-hour surgery, we frequently monitored metabolic parameters, maintaining euvolemia and hemodynamics. Post-extubation, the patient was transferred to the NICU for postoperative monitoring. In the NICU, an increased anion gap of 18 and an elevated beta-hydroxybutyrate level of 0.4 mmol/L were noted, despite normal ABG results and blood glucose levels. Considering perioperative stress and prior SGLT2i use, we initiated intravenous insulin infusion along with IV fluids and electrolyte management according to our NICU DKA protocol to address early-stage euglycemic DKA. His anion gap closed, and beta-hydroxybutyrate levels normalized within the next 12 hours postoperatively. The patient was discharged from the ICU two days later.

## Conclusions

SGLT2i are becoming more prevalent given their utility in the treatment of type 2 DM and heart failure in addition to potential use in weight loss. Euglycemic DKA is a rare but severe adverse effect associated with SGLT2i use. Euglycemic DKA is prone to develop in emergency surgery patients when they are unable to discontinue SGLT2i as a preventive measure, as seen in the documented cases. Diagnosis can be difficult given relative normoglycemia. High clinical suspicion will be needed in the emergency room, ICU, and perioperative settings to make the diagnosis efficiently. Current recommendations include holding SGLT2i for three days before scheduled surgery (four days for ertugliflozin). Preanesthetic evaluations must improve to ensure appropriate management of SGLT2i in the perioperative period.
